# Absolute configuration of (1*R*,3*S*,8*R*,11*R*)-3,7,7,10-tetra­methyl­tri­cyclo­[6.4.0.0^1,3^]dodec-9-en-11-ol

**DOI:** 10.1107/S1600536813018497

**Published:** 2013-07-24

**Authors:** Bimoussa Abdoullah, Auhmani Aziz, My Youssef Ait Itto, Jean-Claude Daran, Auhmani Abdelwahed

**Affiliations:** a Laboratoire de Physico-Chimie Moléculaire et Synthése Organique, Département de Chimie, Faculté des Sciences, Semlalia BP 2390, Marrakech 40001, Morocco; b Laboratoire de Chimie de Coordination, 205 route de Narbonne, 31077 Toulouse Cedex 04, France

## Abstract

The absolute configuration of the title compound, C_16_H_26_O, was determined as (1*R*,3S,8*R*,11*R*) based mainly on the synthetic pathway but is also implied by the X-ray analysis. The mol­ecule contains fused six- and seven-membered rings. Part of the seven-membered ring was refined as disordered over two sets of sites with the occupancy ratio fixed at 0.86:0.14. The disorder corresponds to a major chair conformation and a minor boat conforation. In the crysyal, O—H⋯O hydrogen bonds connect the mol­ecules into chains parallel to the *a* axis.

## Related literature

For related structures, see: Benharref *et al.* (2010 ▶); Gassman & Goman (1990 ▶); Lassaba *et al.* (1997 ▶). For puckering parameters, see: Cremer & Pople (1975 ▶); Boessenkool & Boyens (1980 ▶). For Bijvoet pair analysis, see: Hooft *et al.* (2008 ▶). For analysis of the absolute structure, see: Flack & Bernardinelli (2000 ▶). For chemical properties of related compounds, see: Paresh & Sujit (2012 ▶); Arfaoui *et al.* (2010 ▶). For their biol­ogical properties, see: Chung *et al.* (2007 ▶); Servi *et al.* (2000 ▶). For the synthesis, see: Auhmani *et al.* (2001 ▶).
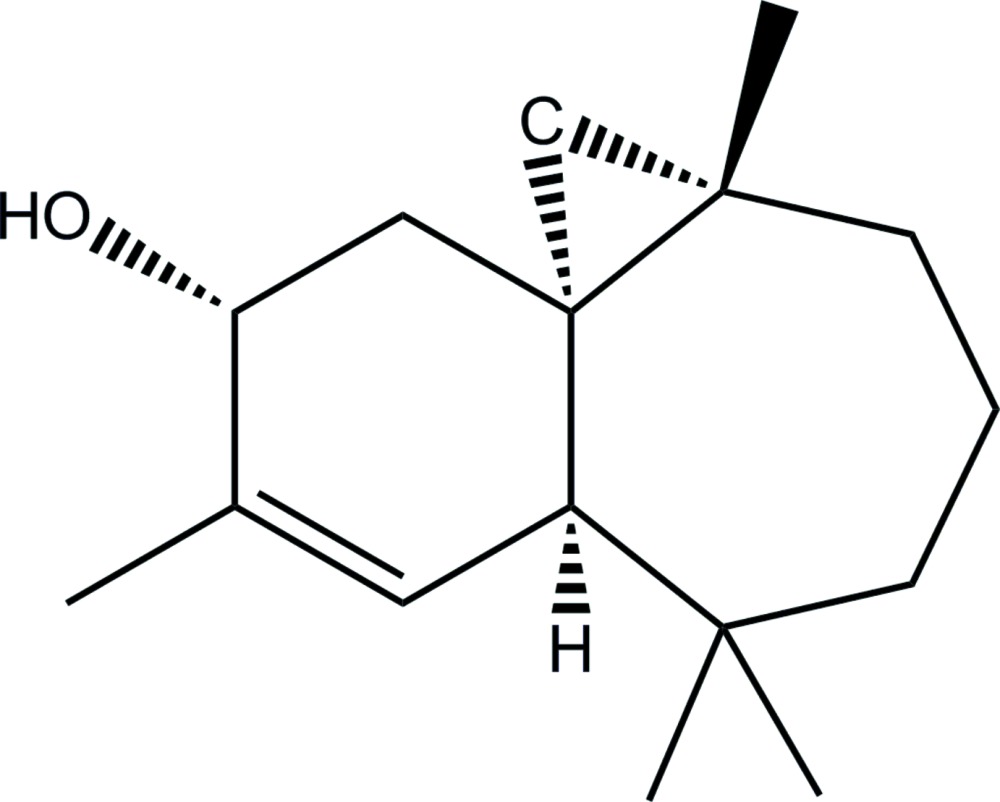


## Experimental

### 

#### Crystal data

C_16_H_26_O
*M* *_r_* = 234.37
Orthorhombic, 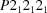

*a* = 6.1457 (1) Å
*b* = 8.2466 (2) Å
*c* = 27.4454 (7) Å
*V* = 1390.96 (5) Å^3^
*Z* = 4
Cu *K*α radiation
μ = 0.51 mm^−1^
*T* = 173 K
0.32 × 0.13 × 0.07 mm

#### Data collection

Agilent Xcalibur (Eos, Gemini ultra) diffractometer
Absorption correction: multi-scan (*CrysAlis PRO*; Agilent, 2012 ▶) *T*_min_ = 0.863, *T*_max_ = 1.000
8172 measured reflections
2653 independent reflections
2539 reflections with *I* > 2σ(*I*)
*R*_int_ = 0.027

#### Refinement

*R*[*F* ^2^ > 2σ(*F* ^2^)] = 0.037
*wR*(*F* ^2^) = 0.098
*S* = 1.04
2653 reflections
190 parameters
29 restraints
H atoms treated by a mixture of independent and constrained refinement
Δρ_max_ = 0.17 e Å^−3^
Δρ_min_ = −0.18 e Å^−3^
Absolute structure: Flack (1983 ▶) 1059 Friedel pairs
Flack parameter: −0.1 (3)

### 

Data collection: *CrysAlis PRO* (Agilent, 2012 ▶); cell refinement: *CrysAlis PRO*; data reduction: *CrysAlis PRO*; program(s) used to solve structure: *SIR97* (Altomare *et al.*, 1999 ▶); program(s) used to refine structure: *SHELXL97* (Sheldrick, 2008 ▶); molecular graphics: *ORTEPIII* (Burnett & Johnson, 1996 ▶), *ORTEP-3 for Windows* (Farrugia, 2012 ▶) and *PLATON* (Spek, 2009 ▶); software used to prepare material for publication: *SHELXL97*.

## Supplementary Material

Crystal structure: contains datablock(s) I, New_Global_Publ_Block. DOI: 10.1107/S1600536813018497/lh5626sup1.cif

Structure factors: contains datablock(s) I. DOI: 10.1107/S1600536813018497/lh5626Isup2.hkl

Supplementary material file. DOI: 10.1107/S1600536813018497/lh5626Isup3.cml

Additional supplementary materials:  crystallographic information; 3D view; checkCIF report

## Figures and Tables

**Table 1:** Hydrogen-bond geometry (Å, °)

*D*—H⋯*A*	*D*—H	H⋯*A*	*D*⋯*A*	*D*—H⋯*A*
O1—H1⋯O1^i^	0.89 (1)	2.32 (1)	3.1612 (6)	159 (2)

Symmetry code: (i) 
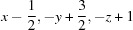
.

**Fig. 1.**
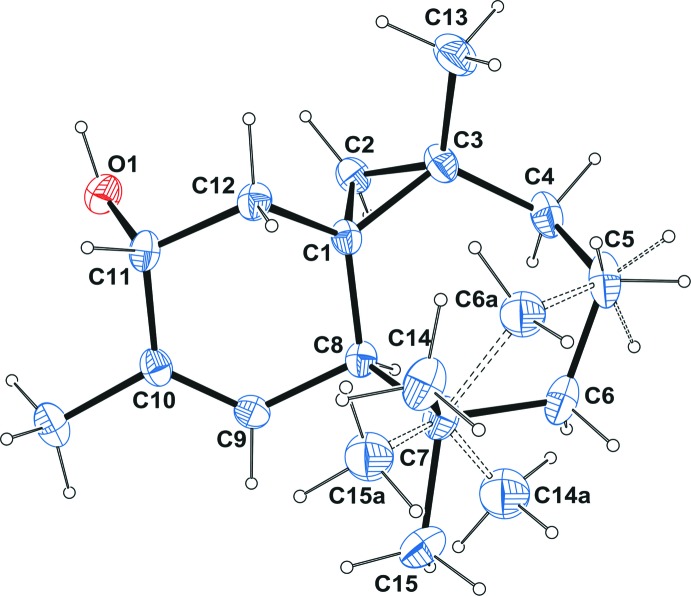
The asymmetric unit of (II) with displacement ellipsoids drawn at the 30% probability level. The dashed bonds represent the minor component of disorder. H atoms are represented as small spheres of arbitrary radii.

**Fig. 2.**
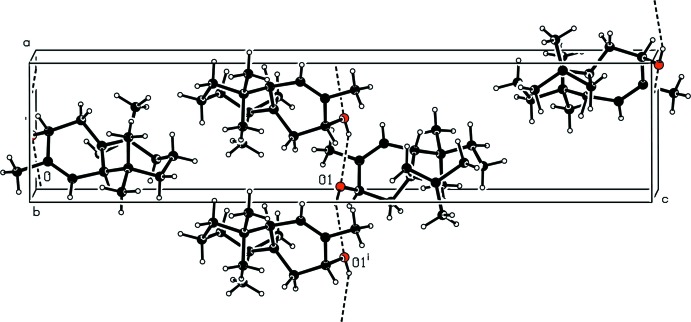
Partial packing view of compound (II), showing the formation of chains parallel to the a axis formed by hydrogen bonds. The minor component of disorder has been omitted for clarity [symmetry code: (i) x - 1/2, -y + 3/2, -z + 1].

## supplementary crystallographic information

### Comment

Optically active allylic alcohols are highly interesting building blocks that have been widely used in organic transformations (Paresh & Sujit, 2012; Arfaoui *et al.*, 2010). Allylic alcohol functionality is also found in several potent biologically active compounds (Chung *et al.*, 2007; Servi *et al.*, 2000). In the aim of preparing chiral allylic alcohols with sesquiterpenic squeleton, we report herein, the crystal structure of the title compound (1*R*,3S,8*R*,11*R*)-3,7,7,10-tetramethyltricyclo[6.4.0.0^1^^,^^3^]dodec-9-en-11-ol (II). The title compound prepared by treating (1*S*,3S,8*R*)-3,7,7,10-tetramethyltricyclo[6.4.0.0^1^^,^^3^]dodec- 9-ene(I) (Auhmani *et al.*, 2001), with *N*-bromosuccinimide (NBS).

The molecular structure of (II) is shown in Fig. 1. As observed in related compounds (Gassman & Goman, 1990; Lassaba *et al.*, 1997; Benharref *et al.*, 2010) the molecule contains a fused six-membered and seven-membered ring. The six-membered ring has approximate half-chair conformation with the puckering parameters: Q = 0.452 (2) Å, spherical polar angle θ= 128.8 (3)° and φ= 153.4 (4)° (Cremer & Pople, 1975), whereas the seven-membered ring displays either a chair conformation (86%) with a total puckering amplitude of 0.797 (3) Å (Boessenkool & Boyens, 1980) or a boat conformation (14%) with a total puckering amplitude of 1.230 (4) Å. The major chair confornation and minor boat conformation corresponds to the disorder in part of the seven-membered ring.

Although the standrad uncertainties on the Flack's parameter (Flack, 1983; Flack & Bernardinelli, 2000), -0.1 (3), and on the Hooft parameter (Hooft *et al.*, 2008), 0.04 (15) are rather high and limit the reliability of the observed value, the absolute configuration (1*R*,3S,8*R*,11*R*) agrees with the one expected from the synthetic pathway. It addition, inverting the configuration leads to values close to 1 for both Flack and Hooft parameters.

In the crystal, the hydroxyl group is engaged in O—H···O hydrogen bonding with symmetry related molecules forming infinite chains parallel to the *a* axis (Fig. 2).

### Experimental

To a cooled (273 K) solution of (I) (4.6 mmol) in 50 ml of a solvent mixture THF/H_2_O (4/1, *v*/*v*), NBS (9,16 mmol) was added in small portions, then mixture was kept under stirring at 273 K, for two hours. After completion of the reaction, a 15% sodium hydrogenocarbonate solution was added and the reaction mixture was taken up in ether, dried over anhydrous sodium sulfate, and concentrated. The crude product was purified by chromatography on silica gel (230–400 mesh) with Hexane/ethyl acetate (96:4) as eluent to give the title compound in 20% yield. X-ray quality crystals were obtained by slow evaporation from a petroleum ether solution of the title compound.

### Refinement

All H atoms attached to C atoms were fixed geometrically and treated as riding with C—H = 0.99 Å (methylene), 0.98 Å (methyl), 0.95 Å (methine) with *U*_iso_(H) = 1.2*U*_eq_(CH and CH_2_) or *U*_iso_(H) = 1.5*U*_eq_(CH_3_). The hydrogen atom of the hydroxyl group was refined with a restraint of O—H = 0.88 (1)Å.

The C6 carbon atom is disordered over two positions inducing a disorder of the two methyl groups C14 and C15 attached to C7. This disorder was modelled using the tools available in *SHELXL97* (Sheldrick, 2008). The two disordered fragment were included in two different parts, PART 1 and 2. The occupancy factor for the two sites was refined using the free variable restraining the sum of the occupancy factors to be equal to 1. The occupancies were ultimately fixed. To be able to calculate the disordered hydrogen atom positions, atom C5 was split in two identical positions which were restrained to have same coordinates and anisotropic thermal parameters by using the EXYZ and EADP instructions in *SHELXL97* (Sheldrick, 2008).

### Crystal data

**Table:**

C_16_H_26_O	*F*(000) = 520
*M**_r_* = 234.37	*D*_x_ = 1.119 Mg m^−^^3^
Orthorhombic, *P*2_1_2_1_2_1_	Cu *K*α radiation, λ = 1.5418 Å
Hall symbol: P 2ac 2ab	Cell parameters from 4275 reflections
*a* = 6.1457 (1) Å	θ = 4.8–70.8°
*b* = 8.2466 (2) Å	µ = 0.51 mm^−^^1^
*c* = 27.4454 (7) Å	*T* = 173 K
*V* = 1390.96 (5) Å^3^	Flattened, colourless
*Z* = 4	0.32 × 0.13 × 0.07 mm

### Data collection

**Table:**

Agilent Xcalibur (Eos, Gemini ultra) diffractometer	2653 independent reflections
Radiation source: Enhance Ultra (Cu) X-ray Source	2539 reflections with *I* > 2σ(*I*)
Mirror monochromator	*R*_int_ = 0.027
Detector resolution: 16.1978 pixels mm^-1^	θ_max_ = 70.9°, θ_min_ = 5.6°
ω scans	*h* = −4→7
Absorption correction: multi-scan (*CrysAlis PRO*; Agilent, 2012)	*k* = −10→10
*T*_min_ = 0.863, *T*_max_ = 1.000	*l* = −33→33
8172 measured reflections	

### Refinement

**Table:**

Refinement on *F*^2^	Secondary atom site location: difference Fourier map
Least-squares matrix: full	Hydrogen site location: inferred from neighbouring sites
*R*[*F*^2^ > 2σ(*F*^2^)] = 0.037	H atoms treated by a mixture of independent and constrained refinement
*wR*(*F*^2^) = 0.098	*w* = 1/[σ^2^(*F*_o_^2^) + (0.0565*P*)^2^ + 0.1685*P*] where *P* = (*F*_o_^2^ + 2*F*_c_^2^)/3
*S* = 1.04	(Δ/σ)_max_ = 0.001
2653 reflections	Δρ_max_ = 0.17 e Å^−^^3^
190 parameters	Δρ_min_ = −0.18 e Å^−^^3^
29 restraints	Absolute structure: Flack (1983) 1059 Friedel pairs
Primary atom site location: structure-invariant direct methods	Flack parameter: −0.1 (3)

### Special details

**Table:**

Experimental. Empirical absorption correction using spherical harmonics, implemented in SCALE3 ABSPACK scaling algorithm. CrysAlisPro (Agilent Technologies,2012)
Geometry. All e.s.d.'s (except the e.s.d. in the dihedral angle between two l.s. planes) are estimated using the full covariance matrix. The cell e.s.d.'s are taken into account individually in the estimation of e.s.d.'s in distances, angles and torsion angles; correlations between e.s.d.'s in cell parameters are only used when they are defined by crystal symmetry. An approximate (isotropic) treatment of cell e.s.d.'s is used for estimating e.s.d.'s involving l.s. planes.
Refinement. Refinement of *F*^2^ against ALL reflections. The weighted *R*-factor *wR* and goodness of fit *S* are based on *F*^2^, conventional *R*-factors *R* are based on *F*, with *F* set to zero for negative *F*^2^. The threshold expression of *F*^2^ > 2σ(*F*^2^) is used only for calculating *R*-factors(gt) *etc*. and is not relevant to the choice of reflections for refinement. *R*-factors based on *F*^2^ are statistically about twice as large as those based on *F*, and *R*- factors based on ALL data will be even larger.

### Fractional atomic coordinates and isotropic or equivalent isotropic displacement parameters (Å^2^)

**Table:**

	*x*	*y*	*z*	*U*_iso_*/*U*_eq_	Occ. (<1)
C1	0.1547 (2)	0.65315 (18)	0.60903 (5)	0.0280 (3)	
C2	0.2298 (3)	0.82761 (18)	0.60418 (6)	0.0343 (3)	
H2A	0.3882	0.8490	0.6054	0.041*	
H2B	0.1469	0.8995	0.5821	0.041*	
C3	0.1203 (3)	0.7732 (2)	0.65035 (6)	0.0355 (4)	
C4	0.2612 (3)	0.7567 (2)	0.69559 (6)	0.0429 (4)	
H4A	0.2338	0.8509	0.7171	0.051*	
H4B	0.4159	0.7607	0.6856	0.051*	
C5	0.2235 (4)	0.6023 (3)	0.72468 (6)	0.0529 (5)	0.86
H51	0.0668	0.5749	0.7238	0.064*	0.86
H52	0.2641	0.6219	0.7591	0.064*	0.86
C6	0.3564 (4)	0.4560 (3)	0.70501 (7)	0.0466 (5)	0.86
H61	0.5115	0.4883	0.7036	0.056*	0.86
H62	0.3446	0.3664	0.7289	0.056*	0.86
C7	0.2906 (3)	0.3898 (2)	0.65441 (6)	0.0385 (4)	
C14	0.0619 (4)	0.3224 (3)	0.65551 (8)	0.0472 (5)	0.86
H14A	0.0520	0.2379	0.6805	0.071*	0.86
H14B	0.0264	0.2758	0.6236	0.071*	0.86
H14C	−0.0409	0.4096	0.6631	0.071*	0.86
C15	0.4476 (4)	0.2429 (3)	0.64537 (9)	0.0511 (5)	0.86
H15A	0.4348	0.1657	0.6723	0.077*	0.86
H15B	0.5978	0.2822	0.6433	0.077*	0.86
H15C	0.4082	0.1892	0.6148	0.077*	0.86
C5A	0.2235 (4)	0.6023 (3)	0.72468 (6)	0.0529 (5)	0.14
H5A1	0.1299	0.6267	0.7531	0.064*	0.14
H5A2	0.3646	0.5614	0.7370	0.064*	0.14
C6A	0.1122 (19)	0.4674 (14)	0.6926 (3)	0.042 (3)	0.14
H6A1	−0.0105	0.5151	0.6741	0.050*	0.14
H6A2	0.0537	0.3808	0.7138	0.050*	0.14
C14A	0.157 (2)	0.2487 (14)	0.6310 (4)	0.044 (3)	0.14
H14D	0.2386	0.2024	0.6037	0.066*	0.14
H14E	0.0173	0.2908	0.6192	0.066*	0.14
H14F	0.1302	0.1645	0.6555	0.066*	0.14
C15A	0.481 (2)	0.347 (2)	0.6806 (6)	0.057 (4)	0.14
H15D	0.4468	0.2601	0.7038	0.086*	0.14
H15E	0.5358	0.4415	0.6983	0.086*	0.14
H15F	0.5928	0.3086	0.6578	0.086*	0.14
C8	0.3284 (2)	0.52177 (18)	0.61381 (5)	0.0282 (3)	
H8	0.4651	0.5796	0.6232	0.034*	
C9	0.3722 (2)	0.44966 (18)	0.56417 (5)	0.0308 (3)	
H9	0.4993	0.3849	0.5610	0.037*	
C10	0.2508 (2)	0.46765 (18)	0.52450 (5)	0.0307 (3)	
C11	0.0428 (3)	0.56358 (18)	0.52503 (5)	0.0331 (3)	
H11	−0.0731	0.4988	0.5084	0.040*	
C12	−0.0336 (2)	0.6044 (2)	0.57649 (5)	0.0314 (3)	
H12A	−0.1401	0.6944	0.5750	0.038*	
H12B	−0.1078	0.5089	0.5907	0.038*	
C13	−0.1071 (3)	0.8335 (3)	0.66163 (7)	0.0502 (5)	
H13A	−0.0981	0.9378	0.6786	0.075*	
H13B	−0.1820	0.7545	0.6824	0.075*	
H13C	−0.1883	0.8473	0.6312	0.075*	
C16	0.3177 (3)	0.3995 (2)	0.47569 (6)	0.0427 (4)	
H16A	0.4620	0.3497	0.4785	0.064*	
H16B	0.3227	0.4870	0.4516	0.064*	
H16C	0.2120	0.3175	0.4653	0.064*	
O1	0.0853 (2)	0.70648 (14)	0.49657 (4)	0.0435 (3)	
H1	−0.046 (2)	0.748 (3)	0.4920 (9)	0.065*	

### Atomic displacement parameters (Å^2^)

**Table:**

	*U*^11^	*U*^22^	*U*^33^	*U*^12^	*U*^13^	*U*^23^
C1	0.0236 (6)	0.0359 (7)	0.0246 (6)	−0.0037 (6)	0.0019 (5)	−0.0029 (6)
C2	0.0342 (7)	0.0337 (7)	0.0349 (7)	−0.0034 (6)	0.0026 (6)	−0.0042 (6)
C3	0.0302 (8)	0.0441 (8)	0.0322 (7)	−0.0030 (7)	0.0042 (6)	−0.0092 (7)
C4	0.0392 (9)	0.0586 (10)	0.0308 (8)	−0.0090 (8)	0.0017 (7)	−0.0121 (7)
C5	0.0572 (11)	0.0760 (13)	0.0256 (7)	−0.0117 (11)	0.0014 (7)	−0.0029 (8)
C6	0.0424 (10)	0.0666 (13)	0.0309 (9)	−0.0071 (11)	−0.0070 (8)	0.0113 (9)
C7	0.0376 (8)	0.0450 (8)	0.0330 (8)	−0.0065 (7)	−0.0021 (6)	0.0080 (7)
C14	0.0421 (10)	0.0557 (12)	0.0436 (11)	−0.0167 (10)	−0.0012 (9)	0.0160 (10)
C15	0.0506 (12)	0.0486 (11)	0.0541 (12)	0.0052 (10)	−0.0055 (10)	0.0171 (10)
C5A	0.0572 (11)	0.0760 (13)	0.0256 (7)	−0.0117 (11)	0.0014 (7)	−0.0029 (8)
C6A	0.051 (7)	0.052 (6)	0.022 (4)	0.000 (6)	0.001 (5)	0.005 (5)
C14A	0.058 (8)	0.041 (6)	0.033 (5)	−0.004 (6)	0.007 (6)	−0.003 (5)
C15A	0.040 (7)	0.065 (9)	0.067 (9)	0.012 (7)	−0.012 (6)	0.010 (7)
C8	0.0222 (6)	0.0355 (7)	0.0269 (7)	−0.0048 (6)	−0.0010 (5)	0.0006 (6)
C9	0.0274 (7)	0.0309 (7)	0.0340 (7)	−0.0005 (6)	0.0040 (6)	−0.0008 (6)
C10	0.0362 (7)	0.0285 (7)	0.0274 (7)	−0.0073 (6)	0.0030 (6)	−0.0025 (6)
C11	0.0335 (7)	0.0388 (8)	0.0269 (7)	−0.0068 (6)	−0.0057 (6)	−0.0010 (6)
C12	0.0226 (6)	0.0411 (8)	0.0305 (7)	−0.0026 (6)	−0.0016 (6)	−0.0002 (6)
C13	0.0388 (9)	0.0636 (11)	0.0481 (10)	0.0056 (9)	0.0103 (8)	−0.0153 (9)
C16	0.0561 (10)	0.0394 (8)	0.0327 (8)	−0.0079 (8)	0.0061 (7)	−0.0080 (7)
O1	0.0504 (7)	0.0459 (7)	0.0343 (5)	0.0059 (5)	−0.0022 (5)	0.0083 (5)

### Geometric parameters (Å, º)

**Table:**

C1—C12	1.516 (2)	C15—H15B	0.9800
C1—C2	1.517 (2)	C15—H15C	0.9800
C1—C3	1.520 (2)	C6A—H6A1	0.9900
C1—C8	1.527 (2)	C6A—H6A2	0.9900
C2—C3	1.503 (2)	C14A—H14D	0.9800
C2—H2A	0.9900	C14A—H14E	0.9800
C2—H2B	0.9900	C14A—H14F	0.9800
C3—C13	1.515 (2)	C15A—H15D	0.9800
C3—C4	1.520 (2)	C15A—H15E	0.9800
C4—C5	1.521 (3)	C15A—H15F	0.9800
C4—H4A	0.9900	C8—C9	1.5106 (19)
C4—H4B	0.9900	C8—H8	1.0000
C5—C6	1.553 (3)	C9—C10	1.328 (2)
C5—H51	0.9900	C9—H9	0.9500
C5—H52	0.9900	C10—C11	1.503 (2)
C6—C7	1.546 (2)	C10—C16	1.510 (2)
C6—H61	0.9900	C11—O1	1.4378 (19)
C6—H62	0.9900	C11—C12	1.526 (2)
C7—C15A	1.421 (11)	C11—H11	1.0000
C7—C14	1.512 (3)	C12—H12A	0.9900
C7—C14A	1.563 (11)	C12—H12B	0.9900
C7—C15	1.568 (3)	C13—H13A	0.9800
C7—C8	1.575 (2)	C13—H13B	0.9800
C7—C6A	1.646 (10)	C13—H13C	0.9800
C14—H14A	0.9800	C16—H16A	0.9800
C14—H14B	0.9800	C16—H16B	0.9800
C14—H14C	0.9800	C16—H16C	0.9800
C15—H15A	0.9800	O1—H1	0.887 (11)
			
C12—C1—C2	115.60 (13)	C7—C14—H14B	109.5
C12—C1—C3	120.38 (13)	C7—C14—H14C	109.5
C2—C1—C3	59.34 (10)	C7—C15—H15A	109.5
C12—C1—C8	113.34 (12)	C7—C15—H15B	109.5
C2—C1—C8	117.89 (12)	C7—C15—H15C	109.5
C3—C1—C8	119.71 (12)	C7—C6A—H6A1	109.7
C3—C2—C1	60.45 (10)	C7—C6A—H6A2	109.7
C3—C2—H2A	117.7	H6A1—C6A—H6A2	108.2
C1—C2—H2A	117.7	C7—C14A—H14D	109.5
C3—C2—H2B	117.7	C7—C14A—H14E	109.5
C1—C2—H2B	117.7	H14D—C14A—H14E	109.5
H2A—C2—H2B	114.8	C7—C14A—H14F	109.5
C2—C3—C13	119.16 (15)	H14D—C14A—H14F	109.5
C2—C3—C4	117.39 (14)	H14E—C14A—H14F	109.5
C13—C3—C4	112.84 (14)	C7—C15A—H15D	109.5
C2—C3—C1	60.21 (9)	C7—C15A—H15E	109.5
C13—C3—C1	119.64 (14)	H15D—C15A—H15E	109.5
C4—C3—C1	118.13 (14)	C7—C15A—H15F	109.5
C3—C4—C5	114.67 (15)	H15D—C15A—H15F	109.5
C3—C4—H4A	108.6	H15E—C15A—H15F	109.5
C5—C4—H4A	108.6	C9—C8—C1	109.06 (11)
C3—C4—H4B	108.6	C9—C8—C7	113.10 (13)
C5—C4—H4B	108.6	C1—C8—C7	116.61 (12)
H4A—C4—H4B	107.6	C9—C8—H8	105.7
C4—C5—C6	112.81 (15)	C1—C8—H8	105.7
C4—C5—H51	109.0	C7—C8—H8	105.7
C6—C5—H51	109.0	C10—C9—C8	126.54 (14)
C4—C5—H52	109.0	C10—C9—H9	116.7
C6—C5—H52	109.0	C8—C9—H9	116.7
H51—C5—H52	107.8	C9—C10—C11	121.90 (13)
C7—C6—C5	116.67 (17)	C9—C10—C16	122.19 (15)
C7—C6—H61	108.1	C11—C10—C16	115.87 (14)
C5—C6—H61	108.1	O1—C11—C10	105.75 (12)
C7—C6—H62	108.1	O1—C11—C12	112.20 (13)
C5—C6—H62	108.1	C10—C11—C12	112.76 (12)
H61—C6—H62	107.3	O1—C11—H11	108.7
C15A—C7—C14	131.7 (7)	C10—C11—H11	108.7
C15A—C7—C6	54.4 (7)	C12—C11—H11	108.7
C14—C7—C6	110.81 (16)	C1—C12—C11	111.64 (12)
C15A—C7—C14A	117.1 (9)	C1—C12—H12A	109.3
C6—C7—C14A	140.3 (5)	C11—C12—H12A	109.3
C15A—C7—C15	51.6 (7)	C1—C12—H12B	109.3
C14—C7—C15	106.92 (17)	C11—C12—H12B	109.3
C6—C7—C15	104.71 (16)	H12A—C12—H12B	108.0
C14A—C7—C15	71.6 (6)	C3—C13—H13A	109.5
C15A—C7—C8	114.2 (7)	C3—C13—H13B	109.5
C14—C7—C8	113.93 (14)	H13A—C13—H13B	109.5
C6—C7—C8	110.66 (14)	C3—C13—H13C	109.5
C14A—C7—C8	107.6 (5)	H13A—C13—H13C	109.5
C15—C7—C8	109.33 (14)	H13B—C13—H13C	109.5
C15A—C7—C6A	108.9 (8)	C10—C16—H16A	109.5
C14—C7—C6A	60.7 (4)	C10—C16—H16B	109.5
C6—C7—C6A	57.7 (4)	H16A—C16—H16B	109.5
C14A—C7—C6A	101.5 (7)	C10—C16—H16C	109.5
C15—C7—C6A	144.2 (4)	H16A—C16—H16C	109.5
C8—C7—C6A	106.2 (4)	H16B—C16—H16C	109.5
C7—C14—H14A	109.5	C11—O1—H1	103.2 (18)

### Hydrogen-bond geometry (Å, º)

**Table:**

*D*—H···*A*	*D*—H	H···*A*	*D*···*A*	*D*—H···*A*
O1—H1···O1^i^	0.89 (1)	2.32 (1)	3.1612 (6)	159 (2)

Symmetry code: (i) *x*−1/2, −*y*+3/2, −*z*+1.
